# The effect of triheptanoin treatment on clinical and laboratory outcomes in patients with long-chain fatty acid oxidation disorder

**DOI:** 10.1007/s00431-025-06216-3

**Published:** 2025-05-31

**Authors:** Engin Köse, Aslı İnci, Havva Yazıcı, Tanyel Zübarioğlu, Sebile Kılavuz, Kısmet Çıkı, Esra Er, Mehmet Cihan Balcı, Ayça Burcu Kahraman, Neslihan Önenli Mungan, Çiğdem Aktuğlu Zeybek, Sema Kalkan Uçar, Leyla Tümer, Fatma Tuba Eminoğlu

**Affiliations:** 1 Division of Pediatric Metabolism, Faculty of Medicine, Ankara University, Ankara, Turkey; 2 Ankara University Rare Disease Application and Research Center, Ankara, Turkey; 3 Division of Pediatric Metabolism, Faculty of Medicine, Gazi University, Ankara, Turkey; 4 Division of Pediatric Metabolism, Faculty of Medicine, Ege University, Izmir, Turkey; 5 Division of Pediatric Nutrition and Metabolism, Cerrahpasa Faculty of Medicine, Istanbul University-Cerrahpasa, Istanbul, Turkey; 6 Division of Pediatric Nutrition and Metabolism, Faculty of Medicine, Marmara University, Istanbul, Turkey; 7 Division of Pediatric Metabolism, Faculty of Medicine, Hacettepe University, Ankara, Turkey; 8 Division of Pediatric Metabolism and Nutrition, University of Health Sciences Dr. Behcet Uz Child Disease and Pediatric Surgery Training and Research Hospital, Izmir, Turkey; 9 Division of Pediatric Nutrition and Metabolism, Faculty of Medicine, Istanbul University, Istanbul, Turkey; 10 Division of Pediatric Nutrition and Metabolism, Faculty of Medicine, Çukurova University, Adana, Turkey

**Keywords:** Creatine kinase, Long-chain fatty acid oxidation disorders, Medium-chain triglycerides, Triheptanoin

## Abstract

Long-chain fatty acid oxidation disorders (LC-FAOD) are a rare metabolic condition that results in impaired fatty acid utilization, leading to metabolic crises, hospitalization, and reduced quality of life. Despite dietary management, many patients experience ongoing complications. Triheptanoin, a seven-carbon triglyceride, has emerged as a therapeutic alternative by providing an energy source and supporting metabolic stability. This study aims to evaluate the clinical outcomes of LC-FAOD patients receiving triheptanoin therapy in Türkiye. A retrospective nationwide study was conducted to analyze 14 patients with LC-FAOD who received oral triheptanoin as part of a compassionate use program in Türkiye. The study collected data on emergency department visits, hospitalizations, metabolic decompensation episodes, creatine kinase (CK) levels, hypoglycemia, and cardiac function. Additionally, patient-reported outcomes were assessed through surveys. The findings of the study demonstrated that triheptanoin treatment led to a significant reduction in the number of emergency service applications and hospitalizations per month (*p* < 0.01). A notable decrease in the frequency of myalgia attacks was observed, while the decline in rhabdomyolysis episodes did not reach statistical significance. Furthermore, creatine kinase levels during metabolic crises exhibited a substantial decrease following triheptanoin therapy (*p* < 0.0001).Among patients with cardiomyopathy, cardiac function showed improvement in four out of seven patients. Survey data indicated an improvement in appetite, physical performance, and overall quality of life.

*Conclusion*: Triheptanoin treatment has been demonstrated to be associated with significant clinical improvements in patients diagnosed with LC-FAOD, including a reduction in the frequency of emergency department visits, hospitalizations, and metabolic crises. These findings provide support for the utilization of triheptanoin as a therapeutic approach that holds promise in the management of LC-FAOD.

**Table:** 

**What is Known:**
• *Long-chain fatty acid oxidation disorders (LC-FAOD) are associated with significant morbidity due to metabolic crises, despite conventional dietary treatment including medium-chain triglycerides (MCT).*
**What is New:**
• *This nationwide study demonstrates that triheptanoin therapy significantly reduces emergency visits, hospitalizations, and creatine kinase levels during crises, and improves patient-reported outcomes, including physical activity and quality of life, in LC-FAOD patients in Türkiye.*

## Introduction

Long-chain fatty acid oxidation disorders (LC-FAOD) are a group of rare autosomal recessive metabolic diseases caused by defects in mitochondrial fatty acid oxidation (FAO). These disorders impair the body’s ability to utilize long-chain fatty acids as an energy source, leading to metabolic decompensation, recurrent episodes of hypoglycemia, rhabdomyolysis, cardiomyopathy, and hepatic dysfunction. The clinical manifestations of LC-FAOD exhibit significant variability, ranging from asymptomatic individuals to severe presentations, including neonatal-onset metabolic crises, exercise intolerance, and life-threatening multi-organ failure [1, 2].

The conventional dietary management of LC-FAOD is primarily focused on the avoidance of fasting, the provision of medium-chain triglycerides (MCTs) as a dietary supplement, and the administration of carbohydrate-rich meals to prevent metabolic decompensation. However, it should be noted that MCTs provide only a two-carbon substrate (acetyl-CoA) for the tricarboxylic acid (TCA) cycle. This may not fully address the metabolic needs of these patients [3]. Triheptanoin, a seven-carbon triglyceride, has emerged as an alternative therapeutic option. Unlike medium-chain triglycerides (MCTs), triheptanoin is metabolized into both acetyl-CoA and propionyl-CoA. This process replenishes TCA cycle intermediates through anaplerotic metabolism, thereby improving energy homeostasis. [4–6].

Several studies have demonstrated that triheptanoin supplementation may reduce the frequency and severity of metabolic crises, decrease hospitalization rates, and improve muscle function in LC-FAOD patients. While some studies report conflicting results regarding CK stability, triheptanoin’s role in crisis management remains promising [7]. Nevertheless, the long-term efficacy of this supplement, particularly in diverse patient populations, remains to be fully elucidated [7–12].

This study aims to evaluate the effects and potential benefits of triheptanoin treatment in patients with LC-FAOD. Additionally, it compares the effectiveness and safety of triheptanoin to MCT oil in a cohort of patients. By providing real-world data, the study seeks to offer valuable insights into the management of LC-FAOD and the role of anaplerotic therapy in enhancing patient outcomes.

## Methods

In this nationwide retrospective study, data were collected from patients diagnosed with Long-Chain Fatty Acid Oxidation Disorders (LC-FAOD). From January 2020 to December 2024, patients received oral triheptanoin treatment as part of the “Compassionate Use Program for Triheptanoin Replacement Therapy in Long-Chain Fatty Acid Oxidation Disorders (UX007)” at eight centers in Türkiye.

The data collated from the medical records of patients encompassed the following: LC-FAOD diagnosis, age at diagnosis, method of diagnosis, age at study enrollment, and details of dietary treatment with MCT oil followed by subsequent triheptanoin therapy. The clinical outcome measures included the number of emergency admissions due to metabolic decompensation and the frequency of hospitalizations per month, both during standard MCT therapy and the subsequent triheptanoin treatment. Creatine kinase (CK) levels were also recorded during and outside metabolic decompensation episodes, as well as any adverse effects observed during treatment. Rhabdomyolysis and myalgia attacks were defined based on clinical symptoms and biochemical findings. Rhabdomyolysis was identified as an episode involving severe muscle pain and/or weakness with a marked elevation in creatine kinase (CK) levels, typically exceeding 1000 U/L (often more than five times the upper limit of normal), and frequently accompanied by systemic signs such as fatigue, dark-colored urine, or the need for hospitalization. In contrast, myalgia attacks were defined as muscle discomfort or pain with mild to moderate CK elevation (generally < 1000 U/L), without systemic manifestations or requirement for inpatient care. The echocardiographic findings of the patients were retrospectively evaluated.

### Survey of quality of life

A survey included questions evaluating various aspects of life, including appetite, weight changes, physical performance, activity levels, sleep quality, mobility, and the ability to travel to evaluate the effects of triheptanoin treatment on daily life. Specific areas assessed in the questionnaire included the following:

#### Appetite

Patients were asked if they experienced any changes in appetite during the treatment, with options to indicate improvement, no change, or worsening.

#### Weight Changes

Patients were asked to report any changes in weight, particularly focusing on weight gain or loss.

#### Physical Performance and Activity

Questions were designed to assess improvements in physical activity levels, including general mobility and the ability to perform daily tasks.

#### Overall Well-being

Patients were asked to rate their overall well-being during the treatment, including perceived improvement in general health.

#### Sleep Quality

Patients were asked about changes in sleep quality, specifically if they had noticed an improvement.

#### Mobility and Travel

The questionnaire assessed improvements in mobility and the ability to travel, with specific attention to any increased ease in movement.

The study was initiated after the approval of the Ethics Committee of Ankara University Faculty of Medicine (İ10–794–24). Written informed consent was obtained from the parents upon enrollment in the study.

### Statistical analysis

Categorical variables are presented as frequencies and percentages, while continuous variables are reported as mean ± standard deviation (range: minimum–maximum). The normality of numerical data was evaluated using the Shapiro–Wilk and Kolmogorov–Smirnov tests. To compare numerical variables, including emergency department visits per month, hospitalizations per month, days per hospitalization, frequency of myalgia episodes, rhabdomyolysis episodes per month, and creatine kinase levels, before and during triheptanoin treatment, either the Wilcoxon signed-rank test or the paired-samples *t*-test was applied. Statistical analyses were performed using IBM SPSS Statistics (Version 26.0, Armonk, NY: IBM Corp.), with a significance threshold set at a two-tailed *p*-value of < 0.05.

## Results

The study included a cohort of patients diagnosed with LC-FAOD who received triheptanoin therapy. Detailed demographic and clinical characteristics, including age, sex, diagnosis, and treatment initiation, are summarized in Table 1.

**Table 1:** Demographic and clinical findings of patients treated with triheptanoin

Parameters	
Current age (month), median [25 th–75 th percentile]	149.7 [68.6–235.2]
Gender (F/M), *n* (%)	7 (50.0)/7 (50.0)
LC-FAOD diagnosis, *n* (%)	
VLCADD	8 (57.2)
CPT2	5 (35.7)
LCHAD	1 (7.1)
Age at diagnosis (month), median [25 th–75 th percentile]	7.6 [1.6–186.1]
Triheptanoin initiation age (month), median [25 th–75 th percentile]	93.5 [49.5–217.7]
Follow-up time before triheptanoin treatment (month), median [25 th–75 th percentile]	44.1 [20.1–80.6]
Follow-up time under triheptanoin treatment (month), median [25 th–75 th percentile]	30.1 [24.5–45.6]
Indication for initiation triheptanoin therapy, *n* (%)	
Rhabdomyolysis	8 (57.1)
Myalgia with elevated CK	5 (35.7)
CMP	4 (28.6)
Hypoglycemia	3 (21.4)
Liver disease	2 (14.3)
Triheptanoin initiation dose (mg/kg/day)/DCI (%), mean ± SD (min–max)	1.4 ± 0.7 (0.5–2.8)/25.4 ± 9.1 (5–39)
Triheptanoin maintained dose (mg/kg/day)/DCI (%), mean ± SD (min–max)	1.7 ± 0.8 (0.3–2.8)/27.7 (7.2–39)
Triheptanoin-related adverse effects, *n* (%)	7 (50.0)
Epigastric pain, nausea	5 (35.7)
Diarrhea	1 (7.1)
Epigastric pain, nausea, diarrhea	1 (7.1)

*CK* creatine kinase, *CMP* cardiomyopathy, *CPT2* carnitine palmitoyltransferase II deficiency, *LC-FAOD* long-chain fatty acid oxidation disorders, *LCHAD* long-chain 3-hydroxyacyl-CoA dehydrogenase deficiency, *max* maximum, *min* minimum, *SD* standard deviation, *VLCADD* very long-chain acyl-CoA dehydrogenase deficiency

The median follow-up period preceding the initiation of triheptanoin treatment was 44.1 months [20.1–80.6], whereas the median follow-up period during triheptanoin therapy was 30.1 months [24.5–45.6]. The most prevalent indications for initiating triheptanoin therapy were rhabdomyolysis (57.1%), myalgia with elevated CK (35.7%), cardiomyopathy (CMP) (28.6%), hypoglycemia (21.4%), and liver disease (14.3%).

The mean initial triheptanoin dose was 1.4 ± 0.7 mg/kg/day (0.5–2.8), corresponding to 25.4 ± 9.1% (5.0–39.0) of the daily caloric intake (DCI), while the mean maintenance dose was 1.7 ± 0.8 mg/kg/day (0.3–2.8), corresponding to 27.7% (7.2–39.0) of the DCI (Table 1).

**Fig. 1:**
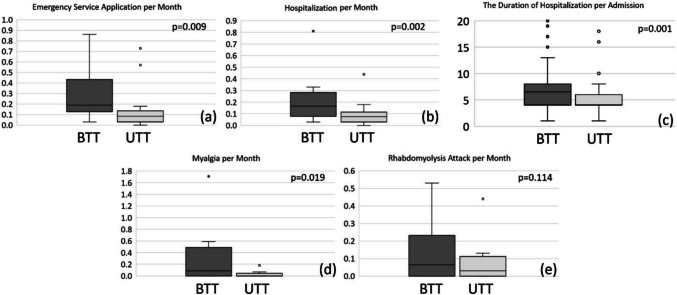
**a** The median number of emergency service applications per month decreased from 0.19 [0.13–0.44] prior to triheptanoin treatment to 0.08 [0.03–0.14] during triheptanoin therapy (*p* = 0.009). **b** The median number of hospitalizations per month decreased from 0.17 [0.08–0.28] before triheptanoin treatment to 0.08 [0.03–0.11] during triheptanoin therapy (*p* = 0.002). **c** The duration of hospitalization per admission also significantly declined following triheptanoin treatment, with the median decreasing from 6 days [4–9] to 4 days [4–6] (*p* = 0.001). **d** A significant reduction in the number of myalgia attacks per month was observed with triheptanoin treatment (0.08 [0.0–0.48] vs. 0.0 [0.0–0.05]). **e** Although a decrease in the number of rhabdomyolysis attacks per month was observed with triheptanoin treatment, this reduction was not statistically significant (0.06 [0.0–0.23] vs. 0.0 [0.0––0.05]). *BTT: before triheptanoin treatment, UTT: under triheptanoin treatment*

The median number of emergency service applications per month decreased from 0.19 [0.13–0.44] prior to triheptanoin treatment to 0.08 [0.03–0.14] during triheptanoin therapy (*p* = 0.009). A similar trend was observed in the median number of hospitalizations per month, which decreased from 0.17 [0.08–0.28] before treatment to 0.08 [0.03–0.11] during therapy (*p* = 0.002). Furthermore, the duration of hospitalization per admission also significantly declined following triheptanoin treatment, with the median decreasing from 6 days [4–9] to 4 days [4–6] (*p* = 0.001). A significant reduction was also observed in the number of myalgia attacks per month with triheptanoin treatment (0.08 [0.0–0.48] vs. 0.0 [0.0–0.05]). Although a decrease in the frequency of rhabdomyolysis attacks per month was noted following triheptanoin therapy, this reduction was not statistically significant (0.06 [0.0–0.23] vs. 0.0 [0.0–0.05]) (Fig. 1).

**Fig. 2:**
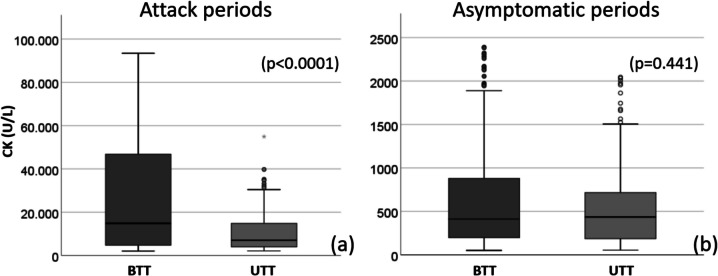
**a** The median creatine kinase level during attack periods decreased from 14,869 [4626–46,851] U/L to 7036 [3955–15,171] U/L following triheptanoin treatment (*p* < 0.0001). **b** The median creatine kinase level during symptomatic periods was 412 [197–881] U/L before triheptanoin treatment, whereas it was 435 [186–715.8] U/L during triheptanoin therapy (*p* = 0.441). *BTT: before triheptanoin treatment, UTT: under triheptanoin treatment*

The median creatine kinase level during attack periods significantly decreased from 14,869 U/L [4626–46,851] to 7036 U/L [3955–15,171] following triheptanoin treatment (*p* < 0.0001). Conversely, the median creatine kinase level during asymptomatic periods exhibited stability, with measurements of 412 U/L [197–881] prior to treatment and 435 U/L [186–715.8] during triheptanoin therapy, showing no statistically significant difference (*p* = 0.441) (Fig. 2).

Three patients (two with CPT2 deficiency and one with VLCADD) had a documented history of hypoglycemic episodes prior to the initiation of triheptanoin treatment. Following the introduction of triheptanoin, a reduction in the frequency of hypoglycemic episodes was observed in all three patients. Notably, none of the patients required hospitalization due to hypoglycemia, either before or after starting triheptanoin therapy.

Three patients with CPT2 deficiency and four with VLCADD presented with CMP. Among them, CMP findings regressed in four patients, while the condition remained stable in three. Notably, none of the patients required emergency service admission or hospitalization due to cardiac involvement either before or during triheptanoin treatment.

Triheptanoin-related adverse effects were reported in 50% of patients, with epigastric pain and nausea being the most common (35.7%), followed by diarrhea (7.1%) and a combination of epigastric pain, nausea, and diarrhea (7.1%) (Table 1). One patient with VLCADD discontinued the treatment at his own request due to these side effects. A female patient diagnosed with CPT2 deficiency, who had a prior diagnosis of epilepsy, unfortunately passed away due to status epilepticus while on triheptanoin treatment. However, there was no evidence of hypoglycemia or metabolic decompensation at the time of the event, and it was not considered to be directly related to triheptanoin use.

A survey of patients and caregivers was conducted to evaluate the effects of triheptanoin treatment on daily life. Triheptanoin treatment exerted a favorable influence on appetite in six out of 14 patients, while concomitantly contributing to weight gain in 10 (71.4%) patients. Additionally, 11 (78.6%) patients reported an improvement in physical performance and activity levels. Furthermore, a positive impact on overall well-being was observed in 13 (92.9%) patients, with nine patients reporting an improvement in sleep quality and 11 (78.6%) patients experiencing enhanced mobility and increased ability to travel.

## Discussion

This pioneering study in Türkiye evaluates the clinical impact of triheptanoin therapy in patients diagnosed with long-chain fatty acid oxidation disorders (LC-FAOD) who are enrolled in the national “Compassionate Use Program for Triheptanoin Replacement Therapy in Long-Chain Fatty Acid Oxidation Disorders.” The study population included 14 patients with LC-FAOD, who received triheptanoin treatment, accounting for 7.2–39.0% of the total daily caloric intake, for a median duration of 30.1 months after switching from standard therapy with MCT oil, which accounted for 8–36% of the total daily caloric intake and was used for a median of 44.1 months. The findings of this study demonstrate that triheptanoin treatment reduced emergency department visits and hospitalizations, while also positively influencing the clinical course of patients with LC-FAOD.

Rhabdomyolysis and elevated CK levels are key clinical manifestations of LC-FAOD, with acute episodes often triggered by infections, fasting, or intense physical activity, leading to emergency visits and hospitalizations. Vockley et al. identified rhabdomyolysis as the most common clinical manifestation in the pretreatment period. While triheptanoin therapy did not result in a significant reduction in hospitalization rates due to rhabdomyolysis, it was associated with a trend toward a 60% reduction in the number of hospital days per year [8]. A similar finding was reported in a study involving three LC-FAOD patients. The study indicated a decrease in hospitalizations related to metabolic crises following treatment with triheptanoin [5]. Yan et al. analyzed rhabdomyolysis, hypoglycemia, and cardiomyopathy in 34 LC-FAOD patients, categorizing these conditions as major clinical events. They found that 36% of patients experienced major clinical events while on triheptanoin compared to 54% while on MCT. These results indicate a lower incidence of major clinical events with triheptanoin treatment. The total mean annualized major clinical event rate was found to be significantly lower with triheptanoin (0.1) compared to MCT (0.7) [13]. Our findings are consistent with the conclusions of prior studies, which indicate that rhabdomyolysis was the most prevalent clinical manifestation before triheptanoin treatment. Triheptanoin therapy has been demonstrated to be associated with a reduction in the frequency of metabolic crises, emergency department visits, and hospitalizations. Furthermore, the median duration of hospitalization per admission exhibited a substantial decline, which may be indicative of an expedited recovery process. Collectively, these improvements provide a robust scientific rationale for the clinical efficacy of triheptanoin as a therapeutic intervention.

A retrospective, nationwide study in Italy encompassing nine patients with LC-FAOD revealed that triheptanoin therapy resulted in a reduction in mean CK levels during metabolic decompensation. Moreover, 77% of patients exhibited lower CK levels outside of metabolic crises in comparison to those receiving MCT oil treatment. While the overall mean CK level was lower during triheptanoin therapy than during MCT oil treatment, the difference was not statistically significant [7]. Conversely, the present study revealed a substantial decrease in CK levels during metabolic crisis episodes, thereby indicating that triheptanoin assists in mitigating muscle damage and enhancing metabolic stability in LC-FAOD patients. Nevertheless, CK levels during asymptomatic periods exhibited relative stability, suggesting that while triheptanoin may not entirely avert CK fluctuations, it may contribute to enhanced metabolic control during crisis periods.

Triheptanoin use was linked to a significant reduction in hospitalizations for hypoglycemia, likely due to its gluconeogenic properties and role in increasing liver glycogen. While this effect was particularly notable in some patients, it must be considered alongside the natural progression of FAOD, where hypoglycemic events are more common in younger individuals and tend to decrease with age. Although aging may have contributed to the observed reduction, the extent of the effect suggests triheptanoin played a key role, especially in certain cases [9, 10]. A 78-week, single-arm, open-label Phase 2 study was conducted to assess the effects of triheptanoin in pediatric and adult patients with severe long-chain fatty acid oxidation disorder (LC-FAOD). A total of 29 subjects were enrolled, and the results suggested that triheptanoin may significantly reduce both the frequency and severity of hypoglycemia in these patients. Treatment with triheptanoin led to a 92.8% reduction in the frequency of hypoglycemic events and a 98.4% improvement in event duration. Before treatment, four patients experienced a total of 12 hypoglycemic events, whereas only one event occurred after starting triheptanoin. Furthermore, prior to treatment, 91.7% of hypoglycemic events resulted in hospitalization, with 16.7% requiring ICU admission. In contrast, the single event during triheptanoin treatment did not require hospitalization [10]. Similarly, in our study, three patients who had experienced hypoglycemic episodes before starting triheptanoin showed a marked reduction in events after treatment. These findings provide substantial evidence to support the potential of triheptanoin in significantly reducing both the frequency and severity of hypoglycemia in LC-FAOD patients.

Cardiomyopathy is one of the most severe complications of LC-FAOD and a leading cause of mortality. A study reported that the mean event rate of cardiomyopathy decreased by 69.6% during triheptanoin treatment, from 0.07 events per year in the pretreatment period to 0.02 events per year during treatment. Additionally, the average annualized duration of cardiomyopathy events was reduced by 75.1%, from 0.60 days per year to 0.15 days following triheptanoin initiation [10]. The LC-FAOD Odyssey study, a non-interventional cohort study, examines the burden of LC-FAOD based on management strategies, clinical event frequency, and healthcare resource utilization using US medical records and patient-reported outcomes. It was reported that while 12% of patients experienced CMP events under MCT treatment, no CMP events were documented under triheptanoin treatment [13]. In a retrospective observational study of an Austrian LC-FAOD cohort, triheptanoin treatment was associated with improvements in cardiac function. Six out of eight patients achieved normalized cardiac function, while two exhibited persistent cardiomyopathy to some extent [9]. In the present study, three patients diagnosed with CPT2 deficiency and four patients diagnosed with VLCADD exhibited CMP. Treatment with triheptanoin resulted in positive improvements in cardiac findings 4 out of 7 patients with CMP. None of the patients required emergency room visits or hospitalization due to cardiac involvement either prior to or during treatment with triheptanoin. These findings demonstrate that triheptanoin treatment may contribute to the improvement or stabilization of cardiomyopathy in patients with LC-FAOD, potentially reducing the risk of cardiac-related complications requiring emergency care or hospitalization.

Triheptanoin is generally well tolerated in LC-FAOD patients, with gastrointestinal side effects being the most commonly reported issue. While some patients experienced treatment-emergent serious adverse events, these are manageable and resolve without long-term complications [6, 11]. In a study involving 94 LC-FAOD patients, triheptanoin-related adverse events were reported in 68.1% of cases, mostly mild to moderate in severity. The most frequently observed side effects included diarrhea, upper abdominal pain, abdominal discomfort, vomiting, and general abdominal pain, with three patients discontinuing treatment due to adverse effects [12]. Similarly, in our study, 50% of patients experienced triheptanoin-related adverse effects, primarily epigastric pain and nausea, with one patient discontinuing treatment due to intolerance. Although one of our patients with a prior diagnosis of epilepsy died due to status epilepticus during triheptanoin treatment, this event was not associated with metabolic decompensation or hypoglycemia and was not considered treatment-related. Notably, a review of the current literature did not reveal any reports of epilepsy or epilepsy-related mortality linked to triheptanoin use.

Individuals diagnosed with long-chain fatty acid oxidation disorders (LC-FAOD) encounter considerable functional impairments, which have a substantial impact on their capacity to engage in routine daily activities, thereby adversely affecting their health-related quality of life. The limitations associated with this condition extend beyond the physical realm, encompassing emotional and psychological well-being, social interactions, and the execution of routine tasks. The presence of recurrent metabolic crises, muscle weakness, fatigue, and exercise intolerance further compels a significant restriction of independence, frequently necessitating the implementation of lifestyle modifications, such as rigorous dietary management and the imposition of activity restrictions. These challenges frequently result in social isolation and psychological distress, underscoring the necessity for comprehensive, patient-centered approaches to care that address both the medical and quality-of-life aspects of the disease [14, 15]. A previous assessment of the effect of triheptanoin on quality of life (QoL) revealed that treatment with triheptanoin significantly improved the daily QoL of patients with long-chain fatty acid oxidation disorders (LC-FAOD). Utilizing a conversion method that transformed QoL measures from Short Form (SF) instruments into EuroQol-Five Dimension (EQ-5D) utility values, the researchers observed a substantial enhancement, with the estimated utility value increasing from 0.365 at the baseline to 0.629 after 78 weeks (*p* = 0.0073) [16]. In our study, the majority of patients reported improvements in appetite, weight gain, physical activity, and overall well-being. Enhanced mobility and the ability to travel more freely highlighted the broader benefits of this therapy, extending beyond just biochemical and clinical outcomes.

There are several limitations to this study. First, the sample size was relatively small, which limits the generalizability of our findings. Additionally, the retrospective nature of the study may introduce bias. Furthermore, we were unable to obtain detailed echocardiographic findings for the patients, which restricts our ability to fully assess the impact of triheptanoin treatment on cardiac function. Future larger, randomized controlled trials are needed to validate our findings and further explore the long-term benefits and safety of triheptanoin treatment.

## Conclusion

This is the first study conducted in Türkiye to evaluate the effects of triheptanoin treatment in patients with LC-FAOD who received oral triheptanoin, revealing significant benefits such as reduced emergency department visits and hospitalizations, improved metabolic stability, and enhanced overall quality of life. While further research is needed, our findings support the use of triheptanoin as a valuable therapeutic option in the management of LC-FAOD.

## Data Availability

No datasets were generated or analysed during the current study.
